# Regenerative Medicine Approaches to Stress Urinary Incontinence

**DOI:** 10.3390/biomimetics11050323

**Published:** 2026-05-06

**Authors:** Alexane Thibodeau, Aiden Smith, Stéphane Chabaud, Geneviève Nadeau, Jean Ruel, Stéphane Bolduc

**Affiliations:** 1 Centre de Recherche en Organogénèse Expérimentale/(LOEX), Regenerative Medicine Division, CHU de Québec-Université Laval Research Center, Quebec, QC G1J 1Z4, Canada; alexane.thibodeau.1@ulaval.ca (A.T.); stephane.chabaud@crchudequebec.ulaval.ca (S.C.); 2 Department of Surgery, Faculty of Medicine, Université Laval, Quebec, QC G1V 0A6, Canada; genevieve.nadeau@fmed.ulaval.ca; 3 Département de Génie Mécanique, Faculté de Sciences et de Génie, Université Laval, Quebec, QC G1V 0A6, Canada; jean.ruel@gmc.ulaval.ca

**Keywords:** stress urinary incontinence, tissue engineering, midurethral sling

## Abstract

Stress urinary incontinence (SUI) affects a significant proportion of women and often requires surgical intervention when conservative treatments fail. While midurethral slings (MUS) are widely used, concerns over complications such as mesh exposure/erosion and chronic pain have driven interest in regenerative medicine alternatives. This review explores emerging strategies, including stem cell therapies, platelet-rich plasma injections, decellularized extracellular matrix scaffolds, injectable hydrogels, and bioengineered slings. These approaches aim to restore continence by promoting tissue regeneration, improving biocompatibility, and reducing adverse reactions. We evaluate their mechanisms, reported outcomes, and current stage of development, supported by in vitro and in vivo model data. Although promising, these technologies face challenges related to cell viability, scaffold integration, and clinical translation. Continued interdisciplinary research is essential to optimize these therapies and bring safer, more effective solutions to patients. Regenerative strategies may ultimately redefine the future of SUI treatment by offering biologically integrated, long-lasting alternatives to synthetic slings. To date, no tissue-engineered or regenerative biomimetic sling has received regulatory approval for routine clinical use in the management of stress urinary incontinence.

## 1. Introduction

Urinary incontinence in women is a prevalent and distressing condition with significant repercussions on physical health, psychological well-being, and social integration. Its impact extends beyond individuals, placing a substantial burden on caregivers and healthcare systems [1,2]. Epidemiological studies estimate the prevalence of urinary incontinence in women to range from 25% to 45%, with stress urinary incontinence (SUI) identified as the most common subtype, which affects approximately 10–20% of the population [3,4]. The development and progression of SUI are influenced by multiple risk factors, including advancing age, multiparity, smoking, and elevated body mass index [5]. The condition is also closely associated with pregnancy and childbirth, which contribute to the weakening of pelvic floor support and increased susceptibility to urinary leakage [5,6,7]. The lifetime risk of requiring at least one surgical intervention for either pelvic organ prolapse (POP) or SUI reaches 20% by the age of 80 [8]. Furthermore, urinary incontinence significantly diminishes the quality of life for these women, with a profound impact on their psychological well-being [9].

Surgical intervention is often required in cases of moderate to severe SUI when conservative treatments fail to provide sufficient symptom relief. In patients with SUI or stress-predominant mixed urinary incontinence (MUI), clinicians may offer the following non-surgical treatment options: continence pessary or vaginal inserts (expert opinion as per AUA Guidelines) [4]. Among available surgical options, midurethral sling (MUS) procedures have become the standard of care due to their efficacy, minimally invasive nature, and relatively low complication rates. These slings function by providing structural support to the urethra, reducing hypermobility and improving urethral closure during episodes of increased intra-abdominal pressure. While synthetic slings, particularly polypropylene-based meshes, are widely used, concerns regarding exposure or erosion, chronic pain, and foreign body reactions have driven interest in alternative approaches. Tissue-engineered biological slings, designed to integrate more effectively with host tissues while minimizing adverse reactions, represent a promising avenue in the evolution of SUI treatment.

Recent comprehensive reviews of urinary system tissue engineering have underscored the rapid expansion of regenerative strategies across urological indications, while emphasizing persistent challenges related to scaffold integration, functional maturation, and clinical translation [10]. Within this broader landscape, the application of regenerative medicine to stress urinary incontinence—particularly through the development of biologically integrated slings—remains an area of active investigation and unmet clinical need.

An integrative conceptual overview of the main regenerative strategies for stress urinary incontinence, including their biological mechanisms and translational pathways, is presented in Supplementary Figure S1.

This literature review aims to provide a comprehensive overview of sling procedures for the surgical management of SUI. It will focus on the development and evaluation of biologically engineered slings created through tissue engineering approaches. By exploring the current advancements, challenges, and clinical potential of these biomaterial-based constructs, this review seeks to highlight their role as emerging alternatives to synthetic and autologous fascial slings in SUI treatment.

## 2. Methodology

This manuscript was designed as a narrative scoping review aimed at synthesizing current regenerative medicine and biomimetics-based strategies for the treatment of SUI, with a particular focus on tissue-engineered slings and adjunctive regenerative approaches.

A comprehensive literature search was conducted in PubMed to identify relevant studies published between January 2015 and February 2025. The search strategy combined the terms “stress urinary incontinence”, “urethral sling”, and regenerative medicine–related keywords, including “tissue engineering”, “regenerative medicine”, “stem cell therapy”, “cell-based therapy”, “biomaterials”, and “bioengineered sling”.

Titles and abstracts were screened for relevance. Studies were included if they:

1. Addressed SUI pathophysiology or treatment;
2. Investigated regenerative, biomimetic, or tissue-engineered approaches;
3. Reported functional, histological, biomechanical, or translational outcomes.

Both in vitro, preclinical in vivo, and early clinical studies were considered. Reviews, opinion pieces, and studies unrelated to continence mechanisms or sling-based support were excluded.

This query yielded a total of 353 articles. Titles and abstracts were screened to assess relevance to the topic of tissue-engineered or regenerative approaches applied to urethral slings for the treatment of stress urinary incontinence. After initial screening, 104 abstracts were retained for in-depth review based on the inclusion of experimental data, preclinical or clinical applications, and relevance to biomaterials or cell-based therapies.

Given the heterogeneous nature of the literature included in this review—encompassing in vitro platforms, multiple small and large animal models, exploratory clinical studies, and case series—formal quantitative risk-of-bias assessment tools (e.g., Cochrane Risk of Bias, SYRCLE) were not uniformly applicable. These instruments are designed for specific study designs and outcomes and do not readily accommodate the diversity of experimental models, endpoints, and translational stages represented in regenerative medicine research for stress urinary incontinence.

Instead, methodological rigor was addressed through explicit qualitative eligibility criteria applied consistently during study selection. Studies were included if they met the following criteria:

(i) direct relevance to stress urinary incontinence pathophysiology or sling-based therapeutic approaches;
(ii) investigation of regenerative, biomimetic, or tissue-engineering strategies;
(iii) reporting of functional, biomechanical, histological, or translational outcomes pertinent to urethral support or continence mechanisms.

Studies were excluded if they lacked relevance to continence mechanisms, did not involve regenerative or biomaterial-based strategies, or were limited to opinion pieces without experimental or clinical data. Methodological quality was, therefore, assessed contextually and narratively, with emphasis placed on experimental design, biological plausibility, outcome relevance, and translational implications rather than on numerical scoring. This approach is consistent with established practices for scoping and translational reviews addressing complex and multidisciplinary research fields.

## 3. Pathophysiology of Urinary Incontinence

### 3.1. Mechanisms of Urinary Continence

**Figure 1:**
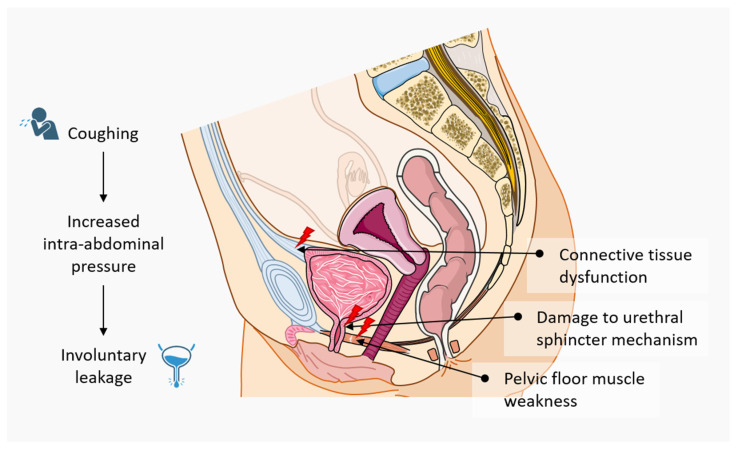
**Pathophysiology of stress urinary incontinence.** Stress urinary incontinence results from impaired urethral support and/or intrinsic sphincter deficiency. Increased intra-abdominal pressure during activities such as coughing, laughing, or physical exertion exceeds the closing pressure of the urethra, leading to involuntary leakage of urine. Key contributing factors include pelvic floor muscle weakness, connective tissue dysfunction, and damage to the urethral sphincter mechanism.

The International Continence Society defines urinary incontinence as the involuntary loss of urine [11]. More specifically, this condition results from dysfunction of the lower urinary tract, which consists of the bladder, urethra, and both the internal and external urinary sphincters. A commonly proposed explanation suggests that stress urinary incontinence results from a combination of disruptions in the supportive connective tissues of the bladder and urethra, as well as a weakening of the muscular structures of the pelvic floor, bladder neck, and urethral sphincters [12,13]. These changes contribute to a reduction in urethral closure pressure and a decrease in abdominal leak point pressure, functionally leading to SUI. When these supportive and muscular components fail to maintain sufficient urethral resistance, urine leakage occurs, particularly during activities that increase intra-abdominal pressure, such as coughing, sneezing, or physical exertion (Figure 1).

### 3.2. Treatment of SUI

Treatments for SUI include conservative, mechanical and surgical interventions. First-line therapy should be behavioral therapies and a decrease in fluid intake [14,15]. Healthy lifestyle habits, such as weight loss, can alleviate the effects of SUI. For example, a study demonstrated that a weight loss of as little as 8% in obese women reduced the occurrence of urinary leakage episodes from 47% to 28% [16]. Conservative treatments also focus on physical interventions aiming to strengthen the pelvic floor muscles [17], including pelvic floor muscle training, vaginal inserts, pessaries [4] and biofeedback [18]. Patients with severe SUI who do not respond to conservative management often require surgical intervention, which generally aims to elevate and support the bladder neck outlet [19]. The primary surgical options for SUI include bulking agents, retropubic suspensions, and autologous and synthetic suburethral slings. However, conservative approaches need to be prioritized before performing a surgical treatment.

## 4. Midurethral Slings: Principles and Limitations

### 4.1. Mesh-Based Midurethral Slings

The purpose of the MUS surgery is to improve the support of the urethra during sudden movements, such as those triggered by coughing or sneezing. This is achieved by elevating and stabilizing the urethrovesical junction using either autologous or synthetic materials. Suburethral sling surgery comprises both synthetic and autologous approaches. Synthetic options consist of midurethral mesh slings implanted either through a retropubic route, as in the tension-free vaginal tape (TVT), or via a transobturator pathway, as in the tension-free vaginal tape–obturator (TVT-O) [4].

Midurethral mesh slings are based on the theory that female SUI results from laxity of the pubourethral ligaments leading to urethral hypermobility; placement of the sling at the midurethra aims to recreate this lost anatomical support. In synthetic MUS procedures, strips of polypropylene mesh are tunneled between the urethra and the anterior vaginal wall and anchored either to the rectus abdominis muscle or to the ileopectineal ligaments [2]. A wide variety of mesh materials and surgical techniques have been described in the literature [19]. Between 2005 and 2013, mesh-based procedures became the most widely performed surgical treatment for female SUI, with approximately 3.7 million meshes sold worldwide during this period [20].

### 4.2. Limitations of Synthetic Slings: Complications and Controversies

Despite evidence from randomized controlled trials demonstrating that synthetic MUS are as effective as traditional surgeries, such as Burch colposuspension and pubovaginal slings, the use of polypropylene mesh for SUI has been surrounded by significant controversy due to its complication profile [21,22]. Most reported complications of standard MUS surgery include overactive bladder (52%), dysfunctional voiding (45%), recurrent or persistent SUI (26%), vaginal mesh exposure (18%), chronic pelvic pain (14%), or local infection (12%) [23]. Chronic pain after MUS surgery occurs in approximately 0.3% to 16% of patients, with most studies suggesting a low prevalence of around 1% [4,24]. However, for patients who do experience pain, the consequences can be significant. While the overall rate appears low, some studies show that for women who do experience pain, it can be life-altering. Nolfi and colleagues further identified a persistent foreign body response within mesh–tissue complexes excised from women who underwent surgical mesh removal months or even years after the initial implantation [25]. Controversies, regulatory warnings, and negative media coverage have increased awareness among both physicians and patients regarding complications associated with vaginal mesh.

The regulation of surgical mesh for SUI varies greatly, reflecting ongoing concerns about its safety. Guidelines highlight the importance of informed shared-decision making, patient education, monitoring complications, and physician training [26]. Ireland, however, implemented a “pause” in 2018 on transvaginal mesh procedures for SUI and POP in cases where safe alternatives exist. Australia implemented a nationwide ban on propylene mesh in 2017, followed by the UK and Ireland in 2018 [27,28]. Nevertheless, several major scientific urological and gynecological associations have published position statements supporting the use of the midurethral sling in the surgical management of SUI [27,29].

Despite its widespread use, uncertainty about surgical mesh persists. Serious complications, such as mesh exposure and chronic pain with complex and morbid surgeries for complete mesh excision, continue to raise concerns, prompting recommendations for the placement and revision of synthetic MUS to be performed only by specialists with thorough follow-up plans [29,30].

## 5. Biological Slings: Characteristics and Benefits

Tissue engineering combines cells, biomaterials, growth factors, and therapeutic agents to address tissue damage or loss. Cells are essential for promoting repair and stimulating the regeneration of affected tissues, while biomaterials provide a supportive scaffold, ensuring structural stability and creating an environment that facilitates the delivery of cells to the damaged site.

Chancellor and colleagues were among the first to explore cell therapy for SUI in a preclinical study focused on intraurethral myoblast injections. This innovative approach aimed to improve urethral wall coaptation and potentially restore urinary sphincter function [31]. Since then, the use of autologous cells for SUI treatment has expanded significantly, inspiring numerous research teams to incorporate cellular components into the design of devices for SUI treatments (as reviewed in [32,33,34]).

Over the years, research has highlighted the significance of dynamic interactions between implanted scaffolds and host tissues in enhancing therapeutic outcomes. This continuous dialogue drives tissue repair and constructive remodeling [35]. Stem cell therapies show promise in the regeneration of the urinary system [32,33,34]. Injection therapy, particularly using adipose-derived stem/stromal cells (ASCs) or muscle-derived stem cells (MSCs), offers promising results in the treatment of SUI, with improvement rates of 88% and 77%, respectively, for intrinsic sphincter deficiency, as highlighted by Barakat and colleagues in a clinical trial review. The therapy demonstrates acceptable functional outcomes with minimal side effects. However, challenges persist despite these promising results. Short-term findings should be approached with caution, as clinical trials have shown inconsistencies and debated conclusions. Despite their safety profile, their clinical efficacy remains limited [36]. In 2022, a research team directly compared the effectiveness of stem cell injection to the MUS procedure. While periurethral injection of MSCs significantly alleviated symptoms with minimal complications in women with SUI, its therapeutic response was notably lower than that of MUS surgery—resulting in full recovery rates of only 33% compared to 80% [37].

A significant limitation of stem cell therapy is the short lifespan of transplanted cells, with survival rates ranging from approximately 8% to 18% within the first weeks post-injection [38]. To optimize the efficacy and longevity of these treatments, further advancements are required to improve cell survival and implantation success. Previous studies have demonstrated that incorporating a hydrogel during cell injection can significantly increase cell viability, with survival rates improving by approximately 30% [39]. These findings suggest that biomaterial-based strategies could play a crucial role in refining stem cell therapies and enhancing their therapeutic potential.

While biomimetic and regenerative approaches offer attractive theoretical advantages over inert synthetic materials, their clinical translation is not without significant challenges. Historical experience with urethral bulking agents highlights these limitations. For example, dextranomer–hyaluronic acid formulations were associated [40,41,42] with local inflammatory reactions at injection sites, while polytetrafluoroethylene injections demonstrated migration concerns, ultimately leading to withdrawal from clinical use. These failures underscore that biocompatibility alone does not guarantee long-term safety or efficacy and emphasize the importance of material stability, tissue integration, and controlled host response.

### 5.1. Decellularized Matrices

Studies have explored the use of decellularized tissues derived from organs such as the urethra and bladder. The primary advantage of these biomaterials lies in their low immunogenicity and their strong potential for neovascularization and smooth muscle cell regeneration [43]. The decellularization process preserves the microstructure and biomechanical properties of the native tissue, making it a promising approach for tissue engineering. Additionally, it may retain the essential biochemical signals of the original tissue, facilitating the repopulation of the urethral extracellular matrix by cells [44,45].

Research has demonstrated that subsequent grafting of these decellularized tissues can facilitate regeneration. Furthermore, these bio scaffolds can later be repopulated with other cell types to generate a “neo-organ.” Regarding sling development, a study conducted in rats found that the reinfiltration of smooth muscle cells (marked by α-SMA) into a decellularized dermis led to a significant recovery of leak point pressure (LPP) [46].

In 2017, a study reported two cases of female urethral reconstruction using an acellular porcine urinary bladder matrix. Acellular biologic grafts, derived from bovine or porcine intestinal mucosa, have a well-established history in reconstructive procedures. After the procedure, the patient remained continent, experiencing no voiding dysfunction, highlighting the potential of such grafts for effective urethral repair [47].

### 5.2. Platelet-Rich Plasma Injections

Platelet-rich plasma (PRP) is an autologous blood-derived product obtained from the patient’s peripheral blood, characterized by a high concentration of platelets and a complex pool of cytokines, chemokines, and growth factors. The bioactive proteins and growth factors within platelet α-granules play a crucial role in key biological processes such as chemotaxis, cellular proliferation and differentiation, angiogenesis, and vascular remodeling [48,49,50]. These regenerative properties make PRP a promising and innovative approach for various medical applications, including urology, where its potential could be leveraged to enhance outcomes in procedures involving urinary slings. Nikolopoulos and colleagues were among the first to propose the use of PRP to treat defects in the pubourethral ligament [51]. Three years later, they demonstrated that the LPP returned to values similar to those observed before the transection of the pubourethral ligament following a PRP injection in female rats [52].

In an initial pilot study, a first report demonstrated the clinical outcomes of PRP in women with SUI. PRP was shown to provide satisfactory clinical outcomes, with local injections leading to noticeable symptom improvement six months post-treatment [53]. Subsequent research further reinforced these findings. In a clinical study by Lee and colleagues, four PRP urethral sphincter injections resulted in 26 patients (93%) experiencing clinical improvement, with 20% achieving complete continence and a pad-free status. The therapeutic effects were significant, appearing immediately after the first injection [54]. These results were further supported by mid-term durability studies, demonstrating that 90% of patients maintained a positive response to treatment over 18 months. Additionally, PRP treatment led to a significant increase in abdominal leak point pressure, indicating improved urethral resistance [55].

While reported clinical improvement rates following PRP injections are encouraging, these outcomes should be interpreted with caution. Most available clinical studies are exploratory in nature, including pilot studies, uncontrolled cohorts, or small case series, and were not powered to support formal statistical inference. Consequently, reported success rates are not consistently accompanied by confidence intervals, comparative statistics, or standardized control groups.

Despite promising preclinical and clinical findings, the translation of PRP therapy for stress urinary incontinence is limited by substantial methodological heterogeneity and a lack of standardization. Significant variability exists across studies in centrifugation protocols, platelet concentration, leukocyte content, activation methods, injection techniques, outcome definitions, and follow-up durations, all of which may influence biological activity and reported clinical outcomes and complicate cross-study comparability [56]. As a result, current PRP outcome data should be interpreted as descriptive indicators of potential efficacy rather than definitive evidence. Establishing standardized PRP preparation, reporting guidelines, and adequately powered controlled trials will be essential to improve reproducibility and enable robust assessment of therapeutic effectiveness.

Beyond clinical trials, in vivo studies have highlighted PRP’s potential in treating pelvic floor dysfunction. Notably, bladder LPP significantly increased in rats after vaginal dilation, suggesting that PRP injections could also play a role in restoring pelvic floor integrity [57]. Autologous myoblast implants resuspended in platelet-poor plasma (PPP) have been explored as a potential treatment for SUI in a rabbit model. The use of PPP as a resuspension medium provided key advantages in the injection process. It stabilized the implant for delivery, ensuring uniform distribution while also minimizing material loss upon needle withdrawal from the urethral wall [58].

More recently, researchers have explored the synergy between PRP and established surgical treatments. A proof-of-concept study investigated the combination of PRP–Fibrin Glue–Stem Cell injections with tension-free vaginal tape (TVT) for mixed urinary incontinence. In this case study, a 56-year-old patient with a seven-year history of mixed urinary incontinence experienced substantial improvement. PRP–Fibrin Glue served as a temporary matrix, stimulating fibroblast proliferation, collagen synthesis, blood vessel formation, and connective tissue regeneration. Following treatment, the patient regained the ability to urinate with an acceptable post-void residue and reported complete satisfaction at a three-month follow-up [59].

These findings suggest that PRP injections may enhance the effectiveness of existing sling procedures, addressing some of the controversies surrounding their long-term efficacy. Nevertheless, PRP-based interventions should still be considered emerging therapies, warranting further rigorous and well-designed clinical studies.

### 5.3. Cell-Free Biomaterials

Quantitative characterization of scaffold properties, including elastic modulus, porosity, and degradation behavior, is essential for understanding material performance and predicting in vivo integration. In the context of regenerative slings for stress urinary incontinence, these parameters are reported across the literature using diverse testing methods and experimental conditions, limiting direct numerical comparison between studies. To address this heterogeneity, scaffold properties in this review are interpreted using representative quantitative ranges and design targets, rather than absolute values.

Across reported biomaterial platforms, candidate sling materials typically exhibit elastic moduli in the range of native pelvic support tissues, generally lower than polypropylene meshes, to reduce stress shielding and erosion risk. Porosity is commonly engineered within an intermediate range that permits cellular infiltration and neovascularization while maintaining sufficient mechanical integrity. Similarly, degradation kinetics are designed to align with tissue remodeling timelines, with gradual resorption occurring over weeks to months, depending on material composition and crosslinking strategy.

Rather than attempting to normalize quantitatively disparate datasets, this review emphasizes how individual studies position their scaffold properties relative to key functional and biological benchmarks, including mechanical compliance, host tissue integration, inflammatory response, and long-term structural stability. This design-principle-based approach allows meaningful comparison of scaffold performance while acknowledging methodological variability in material testing and reporting.

Following implantation, traditional polypropylene synthetic meshes have shown, on histopathological analysis, fibrotic encapsulation and progressive shrinkage or hardening [60]. These effects impair tissue integration, delay wound healing, and can lead to serious complications such as mesh exposure or infection [61]. To overcome these limitations, next-generation urinary slings must be engineered from biomaterials that exhibit not only high biocompatibility but also dynamic mechanical behavior. The optimal material should retain a degree of elasticity to accommodate the forces exerted by routine events such as coughing or sneezing while adapting to strain reversibly, mimicking the dynamic properties of natural, healthy fascia [62].

On the other hand, the biological scaffold material used in tissue engineering must meet several essential criteria to ensure its effectiveness [63]. First, it must exhibit high biocompatibility, allowing for seamless cell adhesion and proliferation while avoiding toxicity or immunogenic responses. Second, it should have an optimal degradation rate, supporting recellularization without compromising structural integrity. Third, sufficient mechanical strength is crucial to support tissue formation while minimizing erosion within soft tissues. Fourth, the material must possess appropriate porosity and pore size, promoting nutrient exchange and cellular infiltration essential for tissue regeneration.

#### 5.3.1. Biocompatibility and Inflammatory Response Mitigation

High biocompatibility is essential to prevent chronic inflammation and ensure long-term implant tolerance. Collagen-based approaches, such as electrochemical compaction and alignment into monofilament threads that can then be woven into meshes for urinary slings, could offer mechanical properties that closely mimic native tissue. These structures support tissue integration, as evidenced by increased collagen deposition and fibroblast proliferation over time, and by a degradation profile compatible with the progression of normal healing. As a natural extracellular matrix component, collagen also contributes to reduced immune activation and improved integration, as shown in long-term implantation studies in large animals [64]. In parallel, electrospun polyurethane slings have also demonstrated superior cytocompatibility compared to conventional polypropylene, while supporting fibroblast adhesion and proliferation [62].

#### 5.3.2. Biodegradation and Remodeling

A well-balanced degradation profile is essential for scaffold remodeling, allowing gradual resorption without compromising the mechanical or structural integrity of the construct. For instance, collagen meshes fabricated via electrochemical alignment support collagen deposition and fibroblast proliferation over time, consistent with a degradation profile aligned with natural healing kinetics [64]. In vivo studies in sheep confirmed that collagen slings maintained their functionality over 12 months, outperforming xenografts that gradually lost their structural integrity [65]. These findings demonstrated the potential of collagen-based urinary slings as durable and mechanically compatible alternatives to conventional graft materials.

Biodegradable polycaprolactone (PCL) meshes have also shown promising remodeling behavior. In a comparative study, MacCraith et al. [66] demonstrated that both PCL and composite meshes offered biocompatibility equivalent to clinically used porcine dermis, while the PCL mesh outperformed all other candidates in promoting collagen production—an indicator of active tissue regeneration. This highlights the capacity of PCL to support constructive remodeling while maintaining an appropriate degradation timeline [66]. In addition, injectable nanofibrillated cellulose hydrogels have shown long-term stability, with 80% retention at injection sites in a canine model after one year, suggesting their potential as durable bulking agents in SUI treatment [67]. Together, these findings emphasize the importance of selecting materials that not only degrade predictably but also actively participate in the remodeling process by supporting cell infiltration and matrix deposition.

#### 5.3.3. Mechanical Properties and Elasticity

Mechanical compliance is another critical design parameter. Materials must provide sufficient strength to support tissue regeneration, while remaining flexible enough to accommodate soft tissue dynamics. Thermoplastic polyurethane meshes, for example, exhibit stiffness values around 0.4 N/mm—significantly lower than polypropylene (2–6 N/mm). This lower stiffness reduces the risk of erosion and provides greater patient comfort [68]. Electrochemically aligned collagen slings also showed apparent modulus comparable to those of native rectus fascia and vaginal tissue, which may reduce the risk of stress shielding and better distribute mechanical loads [65]. Similarly, poly-L-lactic acid (PLA) and PCL scaffolds also show mechanical properties that align with those of vaginal tissues, both in pre- and postmenopausal conditions [69]. It is noteworthy that, in the postmenopausal state, the vaginal mucosa tends to atrophy and thin, increasing the risk of mesh exposure and highlighting the need for a more biocompatible material to reduce such complications [70].

#### 5.3.4. Cell Infiltration and Tissue Integration

Effective scaffold performance relies on the ability to support cellular infiltration, angiogenesis, and nutrient diffusion. This depends on both porosity and surface properties. Electrospun scaffolds, particularly those made of biodegradable PLA or polyurethane, have shown a favorable pore architecture that promotes cell migration and reduces fibrotic encapsulation in animal models [71,72]. An option to overcome erosion involves coating meshes with collagen. Direct tissue contact with polypropylene can promote mesh exposure, while the collagen barrier aims to mitigate this interaction, reducing the mesh exposure rate to 6.4% and potentially improving early integration [73,74].

### 5.4. Cellular Strategies

In addition to acellular scaffolds, cell-based approaches have gained attention for their ability to enhance tissue integration and regeneration through the active contribution of living cells.

#### 5.4.1. Mechanistic Basis of Stem Cell-Mediated Regeneration in Stress Urinary Incontinence

Beyond their potential to differentiate into muscle or stromal cell lineages, stem cells contribute to functional recovery in stress urinary incontinence predominantly through paracrine signaling and immunomodulatory mechanisms. Accumulating evidence suggests that the therapeutic benefits observed following stem cell delivery often outweigh their long-term engraftment or survival, underscoring the central role of indirect regenerative effects [75,76].

Paracrine Signalling Mechanisms

MSCs, ASCs, and muscle-derived stem cells (MDSCs) secrete a broad array of bioactive factors that modulate tissue repair. Key paracrine mediators include vascular endothelial growth factor (VEGF), hepatocyte growth factor (HGF), (basic fibroblast growth factor) bFGF, and insulin-like growth factor-1 (IGF-1). These molecules promote angiogenesis, smooth muscle regeneration, fibroblast activation, and extracellular matrix remodeling, all of which are critical for restoring urethral resistance and pelvic floor integrity [77,78,79].

Immunomodulatory and Antifibrotic Effects

In addition to trophic signaling, stem cells exert significant immunomodulatory effects within the injured urethral and periurethral environment. MSCs have been shown to influence macrophage polarization, favoring a shift from a pro-inflammatory M1 phenotype toward a reparative M2 phenotype. This immunological modulation contributes to attenuation of chronic inflammation, reduces fibrotic encapsulation, and promotes constructive tissue remodeling. Antifibrotic signaling mediated by HGF and related cytokines may further limit excessive collagen deposition, which is a key concern in both synthetic mesh implantation and regenerative scaffold integration [80,81,82].

Cell-Free Regenerative Effects

Emerging evidence indicates that many regenerative benefits traditionally attributed to stem cells can be replicated through cell-free approaches, particularly via extracellular vesicles and exosomes. Stem cell-derived exosomes carry microRNAs, proteins, and signaling molecules capable of recapitulating angiogenic, anti-inflammatory, and pro-regenerative effects without the risks associated with live cell transplantation. Similarly, decellularized ECM derived from stem cell cultures retains bioactive cues that support host cell recruitment, vascularization, and tissue remodeling. These cell-free strategies hold particular translational appeal by reducing regulatory complexity while preserving key regenerative mechanisms [83,84,85].

Collectively, these findings support a paradigm in which stem cell-based interventions for SUI function less as direct tissue replacements and more as biological orchestrators of repair, coordinating vascular, immune, and stromal responses necessary for durable restoration of continence mechanisms.

#### 5.4.2. Mesenchymal Stem Cells as Bioactive Cargo

The integration of MSCs into polymer-based urinary slings represents a promising evolution in sling design, aiming to enhance tissue regeneration, biocompatibility, and long-term functionality. These cell-based strategies harness the paracrine signaling mechanisms and multipotent differentiation capacity of MSCs to support host tissue repair and modulate inflammatory responses. Within this context, both synthetic and natural polymer scaffolds have been investigated as delivery platforms for MSCs, offering distinct mechanical and biological properties suited to different clinical needs.

Synthetic Polymer-Based

Synthetic polymers offer tunable mechanical and degradation properties that make them suitable candidates for engineering urinary slings enhanced with stem cells. Among these, polyglycolic acid (PGA), poly(L-lactide) (PLLA), and PCL have been widely explored for their ability to support mesenchymal stem cell adhesion, differentiation, and matrix deposition.

In a rat model of SUI, ASCs were seeded onto PGA fibers, resulting in strong collagen integration and a significant improvement in leak point pressure, with no signs of infection or exposure [86]. Feng and colleagues further optimized scaffold properties by employing electrospun PLLA/PCL nanofibers seeded with ASCs. The nanofibrous architecture provided a high surface area for cell adhesion, while the addition of estradiol enhanced both the proliferation and myogenic differentiation of rat ASCs [87]. These findings suggest that synthetic slings could be tailored to respond to hormonal environments, potentially offering advantages for postmenopausal patients. Mechanical reinforcement through prolonged in vitro maturation was explored using PGA scaffolds seeded with ASCs and subjected to mechanical loading for 12 weeks. The resulting constructs reached diameters exceeding 2 mm, with densely organized collagen fibers and enhanced mechanical properties. Notably, complete scaffold degradation occurred within this period, enabling full tissue replacement and integration [88]. To further improve the biocompatibility of conventional polypropylene meshes, some studies have examined the incorporation of stem cells. Aslan and colleagues investigated the use of MSCs to mitigate mesh-associated complications, such as pain and exposure [89]. While MSCs did not compromise the mechanical strength of the mesh and may enhance local healing responses, in vivo validation remains necessary. Similarly, Lo and colleagues evaluated human amniotic fluid stem cells (hAFSCs) seeded onto various scaffold materials, and the acellular human dermal matrix (AlloDerm) was the most conducive to cell attachment and proliferation. However, no single scaffold fulfilled all criteria for an ideal implant, highlighting the ongoing need for material optimization [90].

Natural Polymer-Based

Natural polymers are preferred for their close resemblance to native extracellular matrix (ECM) components, which facilitates superior biological integration. Silk fibroin microspheres combined with ASCs have demonstrated the ability to create a supportive three-dimensional microenvironment, promoting cell survival and neo-tissue formation. In a rat model of SUI, it was observed that ASC viability and tissue regeneration up to 12 weeks post-injection, highlighting the scaffold’s potential for durable tissue repair [91]. Similarly, collagen- or fibrin-based injectable microbeads have been investigated as biocompatible carriers for ASCs. These natural microenvironments improved cell retention and facilitated host tissue integration, addressing critical challenges in cell-based therapies [92].

Another promising natural polymer carrier involves chitosan–gelatin hydrogels. Hydrogel slings seeded with MSCs were used in a rat partial sciatic nerve transection model. While the addition of MSCs did not significantly enhance LPP beyond the scaffold alone, its mechanical properties remained stable, suggesting that long-term functional benefits may occur [93].

Combining natural and synthetic polymers, Zhang and colleagues developed a composite nanoyarn scaffold composed of type I collagen and poly(L-lactide-co-caprolactone) [94,95]. Seeded with ASCs and tested in a rat model of vaginal dilation and ovarian resection, this scaffold supported cytoskeletal organization through actin filament expression while mimicking the native ECM structure. Functionally, treated animals exhibited LPP near 40 cmH_2_O, reinforcing its therapeutic potential for pelvic floor repair.

Cell-Based

Three-dimensional cell culture models provide a more physiologically relevant environment, improving cell retention and thus improving their therapeutic potential. The aggregation of ASCs into 3D spheroids has been shown to increase their biological activity [96]. Compared to 2D cultures, 3D microtissues exhibit higher levels of VEGF expression and improve functional recovery in preclinical models of SUI [97]. These microtissue-like structures also promote better cell retention after implantation, a key limitation of cell injection therapies.

Injectable materials incorporating cellular components or their derivatives have also shown promise. A recent strategy involves the use of ECM fragments derived from decellularized ASC sheets as bulking agents. Within one week, these ECM fragments became fully integrated into the surrounding tissue, and by four weeks post-transplantation, host cells had successfully repopulated the injection site [98]. Importantly, ECM retains several bioactive factors secreted by ASCs even after decellularization, including VEGF, bFGF and HGF, which contribute to angiogenesis, immunomodulation, and tissue remodeling. These results highlight the potential of ASC-derived matrices as injectable, bioactive scaffolds with regenerative properties for SUI treatment.

#### 5.4.3. Cell-Attracting Systems—In Situ Recruitment

Research has demonstrated that an injectable formulation composed of cross-linked collagen and fibrin micro-beads, functionalized with bound insulin-like growth factor-1, effectively induces human smooth muscle cell migration in vitro and in vivo. This formulation demonstrated a strong capacity to induce muscle regeneration when injected into the submucosal space of rabbit bladders. Additionally, neovascularization at the injection site was observed, as confirmed by positive CD31 immunostaining [92,99]. Another team developed injectable hydrogels based on β-chitin, which effectively stabilize SDF-1 (stromal cell-derived factor 1) and bFGF within their matrix, ensuring controlled release of these factors. This release facilitates bone marrow stem cells differentiation into fibroblasts. Additionally, the hydrogel’s three-dimensional structure provides a supportive environment for the cells. However, the hydrogel disappeared within 21 days, likely due to degradation or displacement. Nevertheless, the differentiated fibroblasts contribute to the fibroblast population of the anterior vaginal wall, aiding tissue regeneration [100]. The addition of bioactive molecules stimulates local cellular activity, optimizing tissue regeneration.

Another strategy explored in the literature leverages chemokines to direct stem cell migration. Specifically, sulfated proteoglycans have been utilized as carriers for CCL7, facilitating the chemoattraction of MSCs. The response of MSCs to the CCL7 gradient was found to be dependent on their surface receptor profile, underscoring the potential of chemokine-based approaches to enhance cell recruitment in tissue-engineered constructs [101].

In parallel, exosome-based therapies have emerged as a cell-free alternative capable of reproducing some of the paracrine effects of stem cells. In a porcine model of mesh exposure, the local injections of exosomes extracted from peripheral blood stimulated epithelial proliferation and significantly increased capillary density in the regenerating vaginal tissue. A multidose protocol further enhanced these effects, leading to a trend toward thicker epithelium. These findings underscore the regenerative potential of repeated exosome administration in managing chronic complications associated with mesh exposure [102].

## 6. Regulatory Considerations and Clinical Translation

From a regulatory standpoint, regenerative strategies for stress urinary incontinence fall under different classification pathways depending on their cellular and material composition. In the United States, cell-based and tissue-engineered therapies are regulated under FDA 21 CFR Part 1271 as human cells, tissues, and cellular and tissue-based products (HCT/Ps). Products that are more than minimally manipulated, intended for non-homologous use, or combined with biomaterial scaffolds typically fall outside Section 361 exemptions and are instead subject to full biologics or combination-product regulatory pathways.

In the European Union, regenerative therapies are regulated as Advanced Therapy Medicinal Products (ATMPs), including both tissue-engineered products and cell-based therapies, under the authority of the European Medicines Agency. These products require centralized authorization, compliance with Good Manufacturing Practice (GMP) standards, and rigorous long-term safety evaluation. By contrast, purely acellular scaffolds or biomaterials without viable cells may be regulated as medical devices; however, hybrid constructs frequently trigger combination-product classification in both jurisdictions.

Recent translational perspectives further illustrate these challenges. A clinically advanced tissue-engineered approach for stress urinary incontinence based on autologous cells has entered Phase II evaluation, highlighting both the therapeutic promise of regenerative strategies and the substantial regulatory, manufacturing, and logistical hurdles that accompany their clinical development [103].

These regulatory distinctions have important implications for clinical translation, influencing preclinical study design, manufacturing scalability, development costs, and timelines to clinical adoption. Accordingly, regulatory considerations must be integrated early in the development of regenerative slings to ensure alignment between biological innovation, regulatory feasibility, and practical clinical implementation.

Therefore, the clinical translation of tissue-engineered slings, like other tissue-based products, must navigate complex and evolving regulatory landscapes that vary widely across jurisdictions. These products typically fall under the category of ATMPs in the European Union or human cells, tissues, and cellular and tissue-based products (TBP) (HCT/Ps) in the United States, and are often regulated as biologics elsewhere [104]. Because TBPs frequently combine cellular components with biomaterials or devices, they are often classified as combination products, requiring additional scrutiny. Regulatory agencies emphasize safety, reproducibility, and long-term efficacy, often necessitating Good Manufacturing Practice (GMP) compliance and extensive biocompatibility testing. Despite promising early-phase trials, few regenerative therapies for SUI have progressed beyond Phase II. Importantly, a lack of early regulatory planning can delay or prevent clinical translation. Thus, integrating regulatory considerations from the earliest stages of product development is essential to ensure timely and successful market access for tissue-engineered slings.

The scalability and cost-effectiveness of tissue-engineered slings remain critical barriers to widespread clinical use. Autologous cell-based therapies are labor-intensive and costly, while off-the-shelf biomaterials may offer more practical solutions. Manufacturing challenges include batch variability, sterilization, and maintaining cell viability during storage and transport. Studies suggest that tissue-based products can be cost-effective or even cost-saving when total care costs are considered [105]. However, the high initial costs of some products may limit their widespread use [106]. Advances in bioprinting and modular scaffold design may help reduce production costs and improve standardization, facilitating broader clinical implementation.

Ethical considerations are also integral to the clinical translation of regenerative therapies for stress urinary incontinence. These include issues related to informed consent for cell harvesting [37], the use of autologous versus allogeneic cell sources [33,107,108,109,110], equitable patient access to advanced therapies [111], and cost-related disparities [109,112]. Early integration of ethical and societal considerations alongside regulatory planning is essential to ensure responsible development and adoption of regenerative sling technologies.

Despite promising preclinical performance (Table 1) and encouraging early-phase clinical trials, few regenerative therapies for stress urinary incontinence have progressed beyond Phase II evaluation. Barriers to late-stage translation include variability in clinical outcomes, challenges in standardizing cell-based or biologically active products, regulatory complexity, manufacturing scalability, and the need for long-term safety data. As a result, the majority of biomimetic strategies remain confined to experimental or early translational stages [75,76,78].

Importantly, despite significant advances in biomimetics and regenerative medicine, no tissue-engineered sling or regenerative construct has yet been approved for routine clinical use in the treatment of stress urinary incontinence. At present, these approaches remain investigational and should be viewed as future adjuncts or alternatives rather than replacements for established surgical therapies [63,76,113].

Quantitative scaffold properties are reported as representative ranges where available, reflecting design targets rather than directly comparable absolute values owing to methodological heterogeneity. Units, outcome measures, and statistical reporting have been harmonized where possible. When statistical significance or quantitative parameters were not reported in the original studies, this is explicitly indicated to avoid overinterpretation.

## 7. Animal Model vs. In Vitro Model

### 7.1. In Vitro Model

Advancing therapies for SUI requires models that accurately reflect human pelvic anatomy and biomechanics. Small animals such as mice, rats, and rabbits are commonly used in preclinical research, but they have important anatomical differences [114]. In these species, the urethra and vaginal canal are aligned in series, rather than in parallel as in humans. This makes it difficult to test devices that rely on support of the urethra in a human-like configuration. In addition, gravity and abdominal forces act differently on the bladder and urethra in bipedal humans compared to quadrupedal animals. To address these limitations, in vitro models provide a valuable alternative for testing under conditions that closely replicate the human physiological context. Tasmin and colleagues developed an in vitro urinary bladder-urethra model with a synthetic bladder and an artificial urethra to measure the voiding time of the urethra model [115]. This technique faced limitations, primarily due to its lack of complex control over the micturition process. The synthetic bladder was incapable of contraction; urine flow was instead initiated by the backpressure generated from the stored liquid, relying solely on gravitational forces. To provide physiological stimuli and mimic the contractile motion, a syringe pump can be used [116,117]. Recently, Tasmin and colleagues tackled this issue by developing a simplified urinary tract model designed to simulate physiologically relevant pressures [116]. This innovative setup allows the efficacy of a sling to be evaluated in a hammock-like configuration, mirroring its use in human implantation. The bladder model can generate pressures up to 60 cmH_2_O by utilizing a column of stored liquid. It replicates rapid pressure surges that occur during actions such as coughing, where the elevated fluid column induces urine flow through gravitational forces.

Other research groups have adopted simpler techniques to assess the mechanical resistance of slings prior to in vivo grafting [62,118,119]. These methods not only save time but also reduce costs in the development of experimental models, offering an efficient alternative to more complex approaches. Although uniaxial tensile strength (UTS) testing is commonly used to evaluate the mechanical resistance of slings, its two-dimensional analysis fails to accurately represent the three-dimensional forces exerted on the sling within the human body, including the bending forces acting in situ. To address this limitation, a three-point bending test was introduced as an in vitro method to simulate the forces experienced by the sling under physiological conditions [119]. The in vitro three-dimensional force testing model successfully replicates the internal forces acting on the sling within the human body.

The in vitro maturation of tissues under constant strain, also referred to as in vitro mechanical loading or conditioning, has been shown to enhance the mechanical properties of engineered sling tissues over time [88]. However, studies suggest that continuous, unrelenting force may not be the optimal model for replicating physiological conditions. In contrast, dynamic mechanical stimulation (DMS) has demonstrated remarkable potential in improving both the mechanical properties and structural organization of engineered tissues within a bioreactor system.

Gauvin et al. (2011) revealed that applying cyclic strain during tissue development significantly promotes cell reorientation and collagen matrix remodeling [120]. This cellular response enhances the tensile properties of the engineered tissue, increasing both UTS and tensile modulus. Moreover, DMS was found to contribute to the anisotropy of these engineered tissues, reflecting the directional mechanical and structural characteristics inherent to natural tissues. Bioreactors play a pivotal role in replicating the dynamic mechanical environment required for tissue maturation. DMS can simulate physiological mechanical loads and promote the development of stronger, functionally robust, and biologically aligned tissue for sling applications.

While in vitro models provide valuable platforms for early-stage screening of biomaterials and sling designs, they do not fully recapitulate the complex biomechanical, inflammatory, and regenerative environments present in vivo. Consequently, in vitro findings should be interpreted as complementary rather than predictive of clinical performance. Validation through subsequent in vivo models remains essential to assess tissue integration, inflammatory responses, and long-term functional outcomes. Establishing clearer correlations between in vitro mechanical or biological parameters and in vivo or clinical endpoints will be critical to strengthen the translational relevance of these experimental systems.

### 7.2. In Vivo Model

In vivo models are valuable for studying the biomechanical and biological interactions of urinary slings under physiological conditions. These models offer insights into tissue integration, inflammatory responses, long-term durability, and material degradation. By replicating anatomical and mechanical environments, they permit the evaluation of sling efficacy and safety. Animal models mimic pelvic anatomy and simulate mechanical forces experienced during daily activities.

The in vivo model of induced SUI in female rats was first described over 25 years ago [121]. This method used a Foley balloon inflated within the rat’s vagina to simulate birth trauma and studied its effect on the urinary continence mechanism. While the initial trauma caused temporary incontinence, all rats regained continence within one to two weeks, limiting the duration of SUI for study purposes. To extend the duration of SUI, subsequent studies incorporated repeated vaginal balloon dilations (VBD) to prolong the duration of SUI. Pauwels and colleagues conducted a study where rats underwent five consecutive dilations over an eight-week period, ensuring the persistence of SUI throughout the study [122]. This approach proved effective in maintaining incontinence and allowed for the evaluation of long-term effects, including responses to dynamic mechanical stress and tissue remodeling. The sneeze test consistently yielded negative results 14 days after each dilation, thereby confirming the sustained nature of this incontinence model. To further refine the in vivo SUI model, an ovariectomy was incorporated into the experimental protocol. Vaginal balloon dilation and ovariectomy (VBDO) aimed to mimic the hormonal changes associated with menopause, a key factor in the development and persistence of SUI in clinical settings [94,123,124]. The combination of both techniques produces a synergistic effect, resulting in more severe SUI symptoms [125,126]. These models cause urethral epithelial atrophy, loss of serotonergic paraneurons, and alterations in smooth muscle and elastin content [125,127,128].

Over the years, various models have been developed to induce incontinence through different mechanisms, such as pudendal nerve crush or transection [129], electrocauterization [130], and transabdominal urethrolysis [131]. Each method offers specific advantages, for example, studying neurogenic incontinence or intrinsic sphincter deficiency, but they also vary in reproducibility and durability of the induced condition (Table 2).

Bilateral pudendal nerve crush significantly reduces LPP and alters external urethral sphincter electromyography, effectively mimicking SUI symptoms [132,133]. Functional recovery begins approximately two weeks post-injury, with neuroregeneration evident by six weeks [134]. Voiding behavior changes are noticeable within days of injury and gradually normalize as nerve regeneration progresses. Complete transection of the pudendal nerve has been shown to induce the most sustained sphincter deficiency, as evidenced by sphincter muscle atrophy [77,135,136]. Khorramirouz and colleagues developed a model of permanent sphincter deficiency by simultaneously transecting the pudendal nerve trunk and the anastomotic branch of the sciatic nerve [137]. This approach resulted in an irreversible model of urinary incontinence, characterized by irreversible sphincter muscle atrophy. Interestingly, pudendal nerve crush has also been proven to be effective for post-prostatectomy SUI in a male rat model [138].

Urinary incontinence can also be induced through techniques that destroy periurethral tissue, such as electrocauterization or transabdominal urethrolysis. Chermansky and colleagues developed periurethral cauterization as a model of SUI in rats [130]. The procedure is performed via transabdominal access with a transurethral catheter for structural support. Electrocauterization of tissues lateral to the midurethra resulted in a reduction of LPP without impairing bladder function. The effects of the intervention persisted for up to 16 weeks. Urethrolysis has been employed to develop an animal model characterized by lasting urethral dysfunction or reduced urethral resistance. In female rats, transabdominal urethrolysis involves circumferential detachment of the proximal urethra through fascia incision [131]. Following this, the remaining urethra is separated from the anterior vagina and pubis, leading to a notable reduction in LPP that persists for up to 24 weeks post-procedure. Like electrocauterization, this model induces a more prolonged reduction in urethral resistance and causes greater tissue damage compared to childbirth simulation models [139]. It is particularly suited for studying sling procedures.

**Table 2:** Comparison of experimental models used to mimic stress urinary incontinence mechanisms in female animals.

Procedure	Persistence	Application	Advantages/Disadvantages
Vaginal distension	10 days [140]–6 weeks [141]	Childbirth-induced (postpartum) tissue injury	Simple technique ++ Not durable
Vaginal balloon dilation and ovariectomy	8 weeks [125]–9 months [142]	Childbirth-induced (postpartum) tissue injury + aging, Estrogen deficiency	Better reproducing woman’s condition Better persistence
Pudental nerve crush/transection	2–6 weeks [134] 6 weeks [143] 12–18 weeks [46,144] to permanent [137] *	Neuromuscular recovery mechanisms	Fine surgical skills required Selective of neurogenic damage Persistence ++
Electrocauterization	6–16 weeks [130]	Muscular regeneration	Not a phenomenon that occurs naturally in childbirth More significant damage compared to VBD model Simple techniques
Urethrolysis	8–24 weeks [131]	Hypermotility	

* Statement by the team, no evaluation over 4 months.; ++ strength of the study.

Isali and colleagues highlighted the limitations of existing in vivo models for MUS implantation, emphasizing the need for a more representative preclinical model that accurately reflects the suburethral implantation of material in the vaginal wall in humans [145]. Current studies using implantation into the abdominal wall of smaller animal models fail to replicate this specific anatomical and biomechanical environment adequately. Female sheep offer the possibility of suburethral implantation, mimicking the transvaginal tape technique performed in women. Beyond its strong similarity to human physiology, this animal model allows for long-term implantation studies, with follow-ups exceeding one year [65]. Recently, Chapple and colleagues have detailed the technique and ease of use of the ovine model for studying slings designed for women [146]. The main advantage of using sheep is the ability to implant a full-size SUI device, either via a retropubic or suprapubic approach. This model allows the vaginal implantation of materials identical in size and structure to those intended for clinical use. However, it has certain drawbacks, including high costs and the inability to assess the device’s efficacy and comfort. Nevertheless, the surgical technique, including sling tensioning, remains identical to that used in humans. In addition, a team from the University of Tübingen Hospital has developed a model that more accurately reproduces sphincter deficiency [32]. This deficiency is induced in pigs through urethral dilatation combined with electrocautery. However, the use of large animal experiments has explicit limitations. Cohort sizes are often limited, restricting studies to exploratory research. Consequently, interindividual outcomes may rely heavily on a small number of animals.

### 7.3. Conceptual Framework for Normalization and Interpretation of Leak Point Pressure Across Models

Leak point pressure (LPP) is the most commonly reported functional endpoint in preclinical models of stress urinary incontinence. However, absolute LPP values vary widely across studies due to differences in animal species, urethral anatomy, injury paradigms, anesthetic conditions, bladder filling rates, and measurement techniques (Table 3). As a result, direct numerical comparison of absolute LPP values across models or studies is inherently limited.

To improve cross-study interpretability in future research, the field may benefit from adopting normalization-based reporting strategies for LPP outcomes. Such approaches could include expressing LPP changes relative to injury-induced deficits, sham or non-injured controls, within-study comparators, or longitudinal recovery trends.

Widespread adoption of standardized injury models, measurement protocols, and normalized outcome reporting may ultimately facilitate meaningful quantitative synthesis and enable meta-analytic approaches in future translational studies. At present, however, available data remain better suited to qualitative comparative interpretation.

## 8. Discussion

Stress urinary incontinence remains a major clinical challenge, affecting a large proportion of women and often requiring surgical intervention when conservative measures fail. Midurethral slings have become the reference standard due to their effectiveness and minimally invasive nature; however, the complications associated with synthetic meshes—particularly chronic pain, erosion, and foreign body reactions—have highlighted the limitations of purely inert materials. These concerns have catalyzed a paradigm shift toward regenerative medicine approaches that aim not only to provide mechanical support but also to actively restore tissue structure and function.

This review highlights the breadth of regenerative strategies currently under investigation, ranging from cell-based therapies and platelet-rich plasma injections to decellularized matrices, injectable hydrogels, and fully bioengineered slings. Collectively, these approaches seek to improve biocompatibility, promote constructive remodeling, and achieve long-term integration with host tissues. Importantly, emerging evidence suggests that successful sling design depends on more than material selection alone. Scaffold architecture, mechanical compliance, degradation kinetics, and the ability to support cell infiltration and neovascularization are all critical parameters that must be finely tuned to replicate the dynamic biomechanical environment of the pelvic floor.

Cellular strategies, whether based on stem cells, spheroids, or cell-derived extracellular matrices, offer clear regenerative advantages but remain limited by issues of cell survival, delivery, scalability, and regulatory complexity. In contrast, acellular and cell-free biomaterials—particularly collagen-based or biodegradable polymer scaffolds—represent promising “off-the-shelf” alternatives that may strike a more favorable balance between biological performance and clinical feasibility. Hybrid approaches, combining bioactive matrices with endogenous cell recruitment or adjunctive therapies such as PRP, may further enhance tissue regeneration while limiting complexity.

Preclinical evaluation remains a critical bottleneck in the translation of these technologies. While animal models provide valuable insights into biological integration and long-term remodeling, their anatomical and biomechanical differences from humans limit their predictive value. In this context, advanced in vitro models and mechanically relevant testing platforms offer powerful complementary tools to screen materials, optimize scaffold design, and reduce reliance on animal experimentation. The integration of dynamic mechanical stimulation and three-dimensional testing paradigms is particularly important to better replicate the physiological forces experienced by slings in vivo.

When considering the translational potential of regenerative approaches for stress urinary incontinence, it is essential to contextualize these emerging therapies relative to established clinical benchmarks. Midurethral slings remain the current gold-standard surgical treatment, with large clinical series demonstrating high short- and mid-term success rates and relatively low revision rates when performed by experienced surgeons. Nevertheless, synthetic slings are associated with well-recognized complications, including mesh exposure, chronic pain, and the need for complex revision surgery in a subset of patients, which continues to motivate the search for alternative solutions.

In contrast, the regenerative strategies reviewed herein have not yet demonstrated efficacy or durability comparable to midurethral slings and should not be regarded as direct replacements at this stage of development. Rather, these approaches aim to address key limitations of synthetic materials by promoting biological integration, reducing foreign-body responses, and potentially improving long-term safety. Framing regenerative therapies alongside established sling outcomes highlights both the current performance gap and the specific clinical contexts—such as patients at higher risk for mesh-related complications—where biologically integrated solutions may eventually serve complementary or alternative roles.

Long-term safety remains a central consideration for the clinical translation of regenerative strategies. Potential risks include chronic fibrosis, aberrant tissue remodeling, and ectopic tissue formation, particularly for cell-based or bioactive scaffold approaches. Excessive fibrosis may compromise urethral compliance, while uncontrolled remodeling could lead to tissue stiffening or obstruction. Although theoretical concerns regarding tumorigenicity or uncontrolled differentiation have been raised for stem cell–based therapies, available preclinical and early clinical studies have not reported carcinogenic transformation. Notably, the limited long-term survival of transplanted cells in most models suggests that therapeutic effects are predominantly mediated by transient paracrine signaling rather than permanent engraftment, which may mitigate these risks. Nonetheless, extended follow-up remains essential.

Acellular and cell-free strategies—including decellularized matrices, bioresorbable scaffolds, and exosome-based therapies—may offer more favorable safety profiles by avoiding risks associated with persistent living cells. Across all regenerative modalities, systematic long-term evaluation of tissue integration, inflammatory responses, fibrosis, and ectopic tissue formation will be critical for regulatory approval and clinical adoption.

An additional barrier to translation is the lack of standardized functional and patient-centered endpoints across preclinical and clinical studies. Preclinical research relies heavily on leak-point pressure measurements, yet variability in injury models, testing protocols, and reporting practices limits cross-study comparison. Conversely, clinically meaningful outcomes—such as quality-of-life measures, pad tests, and patient-reported outcomes—are rarely captured in animal studies and inconsistently reported in early clinical trials. Addressing this gap will require stage-appropriate standardization, including normalized functional reporting in preclinical models and harmonized continence and quality-of-life endpoints in clinical studies. Such alignment will be essential to improve comparability, support regulatory evaluation, and guide rational assessment of clinical readiness for regenerative approaches to stress urinary incontinence.

## 9. Conclusions

Finally, regulatory, manufacturing, and economic considerations will ultimately determine the clinical adoption of regenerative slings. Early integration of regulatory constraints, standardized manufacturing processes, and cost-effectiveness analyses will be essential to move beyond proof-of-concept studies. While no single regenerative strategy has yet emerged as a definitive replacement for synthetic midurethral slings, the convergence of biomaterials science, tissue engineering, and translational modeling strongly suggests that biologically integrated slings may represent the next generation of surgical treatment for stress urinary incontinence. Continued interdisciplinary research will be key to transforming these promising concepts into safe, durable, and widely accessible clinical solutions.

For practicing clinicians managing patients with stress urinary incontinence today, established therapies, including conservative management, urethral bulking agents with validated safety profiles, and midurethral sling procedures, remain the mainstay of clinical care. Regenerative and biomimetic approaches should currently be considered investigational and are best pursued within structured clinical trials. While these emerging technologies hold long-term promise, patient counseling should emphasize evidence-based treatments currently available, alongside transparent discussion of risks, benefits, and uncertainties.

## Figures and Tables

**Table 1:** Comparative table of the regenerative medicine alternatives to midurethral slings ^1^.

Approach	Key Components	Mechanism of Action	Reported Benefits	Limitations	Stage of Development
Stem Cell Therapy	MSCs, ADSCs	Cell differentiation, paracrine signaling, tissue regeneration	Improved sphincter function, reduced leakage in animal models	Cell survival, delivery method, immune response	Preclinical and early clinical trials TRL 5–6
Platelet-Rich Plasma (PRP)	Autologous platelets and growth factors	Stimulation of tissue repair and angiogenesis	Minimally invasive, promotes regeneration	Variability in preparation, short-term effects	Preclinical and pilot clinical studies TRL 6 (early clinical use)
Decellularized Matrices	ECM scaffolds from porcine or human tissue	Structural support and bioactive cues for host cell infiltration	Biocompatibility, reduced immune response	Batch variability, mechanical strength	Preclinical TRL 4–5
Injectable Hydrogels	Natural/synthetic polymers (e.g., collagen, hyaluronic acid)	Bulking effect, support for cell delivery and integration	Minimally invasive, customizable properties	Degradation rate, mechanical durability	Preclinical TRL 3–4
Bioengineered Slings	Scaffolds seeded with autologous cells or stem cells	Tissue integration and regeneration of supportive structures	Long-term repair potential, reduced foreign body reaction	Complex fabrication, regulatory hurdles	Preclinical and early clinical evaluation TRL 3–5

^1^ To further clarify the maturity of regenerative strategies, approaches were categorized according to Technology Readiness Levels (TRLs). This classification highlights that while PRP and cell injections have entered early clinical application, fully bioengineered regenerative slings remain primarily preclinical, with significant progress required before routine clinical adoption.

**Table 3:** Selected representative preclinical studies of tissue engineering implanted slings in animal models of stress urinary incontinence.

Reference	Method	Animal Model	N	SUI Model	Time Point	Major Histological Findings	Major Functional Findings
Kajbafzadeh, 2015 [46]	Decellularized rat skin	Sprague–Dawley	10	Bilateral pudendal nerve transection	6 wk	α-SMA and CD34 positive. Neovascularization and smooth muscle cell regeneration.	LPP: no significant difference (27.2 ± 5.4 cmH_2_O vs. 27.6 ± 5.9, *p* = 0.832)
Liu, 2023 [57]	Multiple PRP vaginal injections	Sprague–Dawley	8	Vaginal dilation	4 wk	Reduce the expression of MMP-2 and MMP-9. Promote collagen fiber synthesis.	LPP and ALPP significantly increased (*p* < 0.05 and *p* < 0.01)
Shi, 2014 [91]	Silk fibroin microspheres scaffold and ASCs	Sprague–Dawley	9	Proximal sciatic nerve transection	12 wk	ASC survived for 12 weeks post-injection. PGP 9.5 showed the presence of nerve fibers around urethral muscles.	LPP: recovered (59.74 ± 7.88 cmH_2_O, *p* < 0.05)
Wang, 2016 [86]	ASCs seeded on polyglycolic acid fibers	Sprague–Dawley	5	Vaginal dilatation and bilateral ovariectomy	8 wk	Masson staining: increased expression of collagen fibers.	LPP: significantly increased (35.96 ± 1.87 cmH_2_O, *p* > 0.05)
Zhang, 2018 [94]	Type 1 collagen and PLCL cultured with ASC	Rat	6	Vaginal dilatation and bilateral ovariectomy	8 wk	Cells infiltrated into majority of nanoyarn, and only a little part of naked nanoyarn could be found. Sufficient mechanical strength	LPP: 38 cmH_2_O
Pinar, 2022 [147]	umbilical vessel sling	rat	10	Bilateral pelvic nerve injury	4 wk	Good local tolerance and a moderate to high tissue integration	LPP: increased but not statistically significant (21.8 ± 2.1 mmHg vs. 28.4 ± 4.1 mmHg, *p* = 0.2) Micturition frequencies: similar Total voided volume: higher (12.5 ± 1.1 mL vs. 9.4 ± 0.6 mL, *p* < 0.05)
Naji, 2024 [93]	Chitosan–gelatin hydrogel loaded with MSC	Sprague Dawley	6	Proximal sciatic nerve transection	3 wk	Inflammation rate was significantly lower in chitosan–gelatin hydrogel loaded with MSC group	LPP = No significant difference

LPP: leak point pressure, ASC: adipose-derived stem cells, PLCL: Poly (L-lactide-co-caprolactone), MSC: muscle-derived stem cells, wk: week.

## Data Availability

No new data were created or analyzed in this study. Data sharing is not applicable to this article.

## Supplementary Materials

The following supporting information can be downloaded at: https://www.mdpi.com/article/10.3390/biomimetics11050323/s1, Figure S1: Conceptual overview of regenerative strategies for stress urinary incontinence.
