# Supplementary material for: Regenerative Medicine Approaches to Stress Urinary Incontinence
Source: Biomimetics (Basel). 2026 May 6;11(5):323. doi: 10.3390/biomimetics11050323 (PMC13204115; doi:10.3390/biomimetics11050323)
Supplement: Supplementary file 1 [file biomimetics-11-00323-s001.zip › biomimetics-4228459-supplementary.pdf]

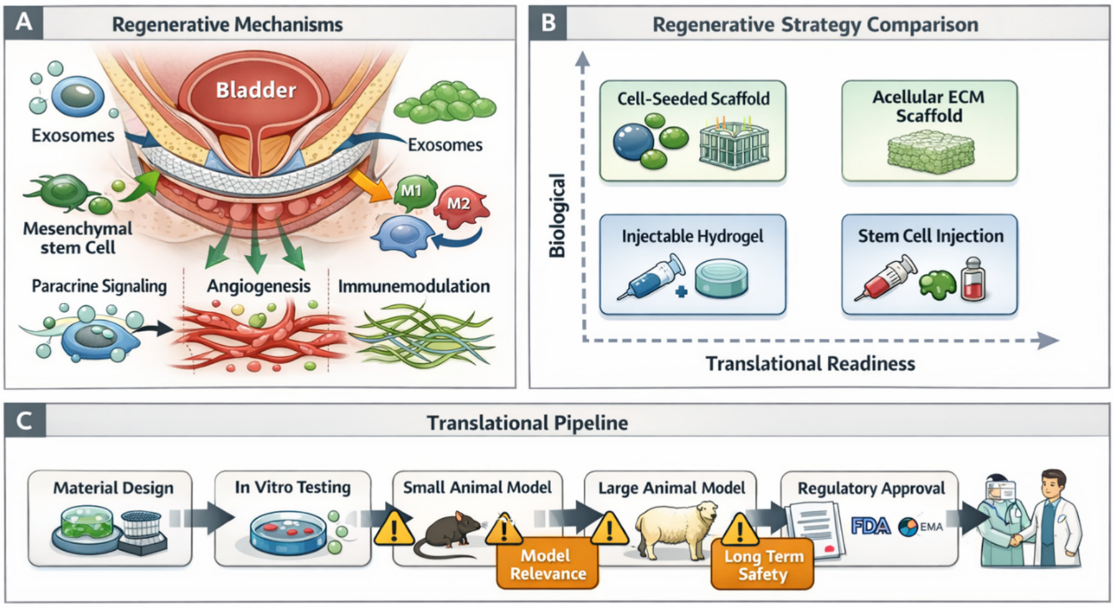

Figure S1. Conceptual overview of regenerative strategies for stress urinary incontinence. (A) Mechanism based schematic illustrating key biological pathways targeted by regenerative approaches, including paracrine signaling, immunomodulation, angiogenesis, and extracellular matrix remodeling. (B) Comparative framework positioning regenerative strategies according to biological complexity and translational readiness. (C) Translational pipeline highlighting key stages and bottlenecks in the development of regenerative slings from bench to clinical application. (Microsoft 365 Copilot (licensed Microsoft 365 Copilot experience embedded in Microsoft 365 applications, November–December 2025 release).
